# From Growth Trajectory to Functional Decline: Age-Contextualized Nutritional Strategies for Muscle Vulnerability. A Narrative Review

**DOI:** 10.3390/nu18091437

**Published:** 2026-04-30

**Authors:** Luisa Malaguarnera, Vincenzo Sortino, Sofia Surdo, Salvatore Piro

**Affiliations:** 1 Department of Clinical and Experimental Medicine, University of Catania, 95123 Catania, Italy; luisamalaguarnera92@gmail.com (L.M.); salvatore.piro@unict.it (S.P.); 2 PhD Program in Innovative Technologies in Biomedical Sciences, University Kore of Enna, 94100 Enna, Italy; 3 National Council of Research, Institute for Research and Biomedical Innovation (IRIB), Unit of Catania, 95123 Catania, Italy; 4 Assotiations ABC OdV Catania, 95027 Catania, Italy; s.surdo98@yahoo.com

**Keywords:** skeletal muscle, pediatric growth, geriatric sarcopenia, anabolic resistance, impaired muscle accretion, protein density, inflammaging, age-contextualized nutrition

## Abstract

Muscle vulnerability occurs at both extremes of the human lifespan, although its biological significance differs substantially between developmental growth and late-life decline. During childhood and adolescence, insufficient muscle accretion reflects disruption of physiological anabolic trajectories driven by inadequate energy availability, inflammatory burden, endocrine imbalance, or disease-associated catabolism. In older adults, muscle deterioration is characterized by anabolic resistance, neuromuscular remodeling, chronic low-grade inflammation, and hormonal decline, culminating in sarcopenia and loss of functional independence. The absence of harmonized diagnostic frameworks across age groups limits direct translational extrapolation. A lifespan-informed perspective distinguishing growth-supportive from function-preserving nutritional approaches is, therefore, required. This narrative review examines how major classes of nutritional bioactive interact with molecular pathways regulating skeletal muscle homeostasis in fragile populations across the lifespan. The analysis encompasses energy adequacy, protein quantity and quality, amino acid-dependent anabolic signaling, vitamin D status, lipid-derived mediators, redox-modulating phytochemicals, and micronutrients supporting mitochondrial bioenergetics. In pediatric contexts, nutritional interventions primarily aim to restore anabolic permissiveness within a structurally intact growth environment. In aging individuals, strategies focus on mitigating anabolic resistance through optimized protein intake, correction of micronutrient insufficiencies, and integration with resistance exercise to preserve functional capacity. This narrative review emphasizes the need to distinguish mechanistic rationale from clinically validated interventions, as improvements in molecular pathways do not consistently translate into meaningful functional outcomes.

## 1. Introduction

Skeletal muscle is a dynamic metabolic and endocrine tissue that plays a central role in locomotion, glucose homeostasis, protein storage, immune modulation, and maintenance of functional independence. Across the human lifespan, muscle mass and performance are shaped by the interaction of nutrient availability, endocrine regulation, inflammatory signaling, mitochondrial integrity, and neuromuscular activity [1].

Disruption of these regulatory networks contributes substantially to morbidity at both extremes of life, although the biological nature of muscle vulnerability differs profoundly between developmental growth and aging-related decline [2]. Central nodes within this regulatory architecture include the insulin– insulin-like growth factor 1 (IGF-1)–Protein kinase B (AKT) pathway, the mechanistic target of rapamycin complex 1 (mTORC1), and energy-sensing mechanisms governed by AMP-activated protein kinase (AMPK). Together, these pathways coordinate translational initiation, mitochondrial metabolism, autophagic turnover, and cellular stress responses, thereby maintaining muscle protein homeostasis [3]. Although these signaling systems are conserved across the lifespan, their functional context differs markedly between developmental growth and aging. During childhood and adolescence, skeletal muscle develops within a highly anabolic physiological environment sustained by growth hormone (GH)-IGF-1 signaling, pubertal endocrine maturation, and adequate energy and protein availability. Muscle accretion is closely coupled to linear growth and reflects coordinated processes of myofiber expansion, satellite cell activation, and neuromuscular maturation [4]. When this developmental trajectory is disrupted due to chronic inflammatory disease, congenital conditions, oncologic therapies, gastrointestinal disorders, malnutrition, or prolonged immobilization, the result is impaired lean mass accrual relative to expected growth patterns rather than true degenerative muscle loss. Importantly, diagnostic constructs developed for adult sarcopenia cannot be directly applied to pediatric populations because body composition, muscle strength, and neuromuscular function undergo continuous age-dependent changes during growth [4]. In contrast, muscle decline in older adulthood reflects progressive impairment of anabolic signaling combined with structural and metabolic remodeling of skeletal muscle tissue. Aging muscle is characterized by reduced responsiveness to anabolic stimuli, impaired neuromuscular function, mitochondrial dysfunction, chronic low-grade inflammation, and endocrine alterations including reductions in sex steroids, GH, and IGF-1. These processes converge in the clinical syndrome of sarcopenia, defined by loss of muscle mass, strength, and physical performance, which is strongly associated with frailty, falls, disability, metabolic dysregulation, and increased mortality [5]. Despite these fundamental biological differences, nutritional strategies aimed at preserving muscle health are frequently discussed within a unified anti-sarcopenic framework. However, extrapolating interventions developed for geriatric sarcopenia to pediatric fragile populations may be biologically inappropriate and clinically misleading [6]. In this review, the term fragile populations refers to individuals with increased vulnerability to impaired skeletal muscle homeostasis due to developmental, clinical, or metabolic factors. In pediatric contexts, this vulnerability is primarily associated with disrupted growth conditions, including undernutrition, chronic disease, or endocrine imbalance. In older adults, it reflects age-related declines in anabolic responsiveness, often compounded by frailty, multimorbidity, and functional impairment.

In older adults, nutritional interventions are primarily designed to counteract anabolic resistance through higher protein density, optimized amino acid composition, strategic nutrient timing, and integration with resistance exercise. In pediatric contexts, by contrast, the priority is to restore physiological growth conditions by ensuring adequate energy intake, safe protein provision, micronutrient sufficiency, and stabilization of inflammatory and endocrine disturbances [7]. Beyond macronutrient sufficiency, increasing evidence indicates that bioactive dietary compounds influence intracellular signaling networks regulating muscle protein turnover, mitochondrial function, oxidative stress, and inflammatory responses [8]. Amino acids activate nutrient-sensing pathways that control translational efficiency, lipid-derived mediators modulate inflammatory tone and metabolic flexibility, vitamin D participates in endocrine and redox regulation, while polyphenols and micronutrients contribute to mitochondrial resilience and cellular homeostasis. The biological impact of these factors is highly dependent on developmental stage, baseline anabolic sensitivity, and the integrity of neuromuscular and metabolic regulatory systems [9]. A lifespan-informed perspective is, therefore, required to interpret nutritional interventions targeting muscle vulnerability [10]. Rather than applying a uniform anti-sarcopenic paradigm, strategies should be contextualized within the distinct molecular and physiological environments that characterize pediatric growth and aging muscle decline [11].

Importantly, although the molecular mechanisms underlying skeletal muscle regulation are increasingly well characterized, their translation into clinically meaningful outcomes remains heterogeneous across nutritional interventions. In many cases, activation of anabolic or metabolic pathways does not necessarily result in measurable improvements in muscle strength, physical performance, or functional independence. In this review, we therefore explicitly distinguish between: (i) mechanistic evidence derived from cellular and animal models, (ii) observational and physiological data in humans, and (iii) interventional clinical evidence demonstrating effects on clinically relevant endpoints. This distinction is essential to avoid overinterpretation of biologically plausible mechanisms that are not consistently supported by clinical benefit and to provide a more balanced and translationally grounded interpretation of current evidence.

By comparing pediatric growth-related vulnerability with aging-associated sarcopenia, this review aims to integrate mechanistic insights with clinical evidence, highlighting how differences in anabolic signaling, mitochondrial function, inflammatory regulation, and metabolic flexibility shape the effectiveness of nutritional interventions across the lifespan.

### Literature Identification and Selection Strategy

Although this work is presented as a narrative review, particular attention was given to ensuring transparency in the identification and selection of the literature. Relevant studies were identified through searches in major biomedical databases, including PubMed/MEDLINE and Scopus, with a focus on publications addressing skeletal muscle metabolism, sarcopenia, pediatric muscle development, and nutritional bioactives. Priority was given to recent studies and, where available, to human data, including clinical trials and observational studies reporting outcomes related to muscle mass, strength, and physical performance. Mechanistic studies from cellular and animal models were included to provide biological context, particularly in areas where clinical evidence remains limited. The selection of studies was guided by relevance to the central theme of age-contextualized muscle vulnerability and by their contribution to understanding the interaction between nutritional factors and molecular pathways regulating muscle homeostasis.

## 2. Pediatric Muscle: Growth-Oriented Anabolic Environment

During childhood and adolescence, skeletal muscle develops within a physiologically anabolic framework sustained by active growth hormone IGF-1 signaling, preserved insulin sensitivity, and high translational efficiency. These conditions support rapid myofiber hypertrophy, longitudinal muscle growth, and continuous remodeling of the neuromuscular system. Satellite cells play a central role in this process by providing additional myonuclear required for fiber expansion and by contributing to regenerative capacity during periods of rapid tissue development [4].

Nutrient availability is tightly coupled to these growth processes. Adequate energy intake maintains cellular adenosine triphosphate (ATP) levels required for protein synthesis, while amino acids activate nutrient-sensing pathways that stimulate mTORC1-dependent translational initiation. Under physiological conditions, this signaling environment enables efficient conversion of dietary substrates into structural muscle proteins, supporting coordinated increases in muscle mass and strength during growth [12]. In pediatric populations, muscle vulnerability typically arises not from intrinsic degenerative processes but from disruption of this anabolic trajectory. Chronic inflammatory diseases, congenital disorders, prolonged immobilization, oncologic therapies, or insufficient nutrient intake can interfere with endocrine signaling and nutrient-dependent translation. Inflammatory cytokines such as tumor necrosis factor-α (TNF-α) and interleukin-6 (IL-6) may suppress insulin receptor substrate signaling, reduce AKT activation, and promote transcription of proteolytic genes through nuclear factor kappa B (NF-κB) and Forkhead box O (FOXO)-dependent pathways. These mechanisms limit muscle accretion relative to developmental expectations rather than inducing primary muscle degeneration [13]. Importantly, the molecular framework responsible for anabolic signaling remains largely intact in pediatric muscle. Consequently, restoration of adequate energy intake, correction of micronutrient deficiencies, and control of inflammatory burden can often re-establish physiological anabolic signaling and support recovery of growth-aligned muscle development.

## 3. Aging Muscle: Anabolic Resistance, Proteostatic Imbalance, and Mitochondrial Dysfunction

**Figure 1:**
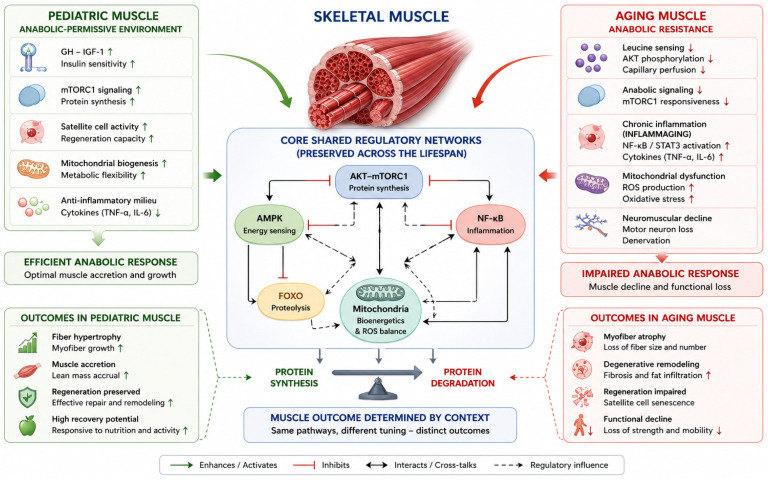
Skeletal muscle regulation across the lifespan. Skeletal muscle homeostasis is governed by conserved molecular pathways integrating anabolic signaling, energy sensing, inflammation, and mitochondrial function. Core regulatory networks including AKT–mTORC1, AMPK, NF-κB, FOXO, and mitochondrial bioenergetics are shared across life stages and centrally determine the balance between protein synthesis and degradation. In pediatric muscle, a physiologically anabolic-permissive environment characterized by active GH-IGF-1 signaling, preserved insulin sensitivity, and satellite cell activity supports efficient activation of these pathways, promoting muscle accretion, fiber hypertrophy, and regenerative capacity. Transient inflammatory signals may inhibit anabolic signaling but do not compromise the intrinsic regenerative potential. In aging muscle, the same regulatory networks are progressively dysregulated. Reduced leucine sensing, impaired AKT phosphorylation, chronic low-grade inflammation (inflammaging), mitochondrial dysfunction, and neuromuscular decline collectively contribute to anabolic resistance, increased proteolysis, and myofiber atrophy. Overall, muscle phenotype is determined by context-dependent modulation of shared pathways rather than by condition-specific mechanisms, resulting in distinct functional outcomes across the lifespan. Abbreviations: AKT, protein kinase B; FOXO, Forkhead box O; GH, growth hormone; IGF-1, insulin-like growth factor-1; IL-6, interleukin-6; IRS, insulin receptor substrate; mTORC1, mechanistic target of rapamycin complex 1; MuRF1, muscle RING finger protein 1; PI3K, phosphoinositide 3-kinase; TNF-α, tumor necrosis factor alpha.

In older adulthood, the biological context of skeletal muscle regulation changes substantially, resulting in reduced capacity to translate anabolic stimuli into effective protein synthesis, a phenomenon commonly described as anabolic resistance. This impaired responsiveness arises from multiple mechanisms, including reduced amino acid sensing, diminished AKT phosphorylation, altered intracellular trafficking of mTORC1, and decreased ribosomal biogenesis [14]. Simultaneously, aging muscle undergoes progressive structural and metabolic remodeling characterized by mitochondrial dysfunction, reduced oxidative capacity, and increased production of reactive oxygen species. Chronic low-grade inflammation, commonly termed “inflammaging”, further interferes with insulin and IGF-1 signaling pathways while promoting activation of catabolic transcriptional programs [15]. Neuromuscular alterations, including progressive motor neuron loss and incomplete collateral reinnervation, reduce motor unit number and contribute to declines in muscle strength and coordination. Age-related reductions in circulating sex steroids, GH, and IGF-1 further weaken anabolic signaling networks and exacerbate resistance to nutrient-derived stimuli [16]. In parallel, disruption of the balance between protein synthesis and cellular quality control mechanisms contributes to progressive proteostatic decline. Defective autophagic flux and impaired mitochondrial turnover lead to accumulation of damaged organelles and oxidized proteins, reinforcing metabolic inefficiency and oxidative stress. Persistent activation of energy-sensing pathways may further suppress mTORC1 activity, thereby limiting regenerative capacity and contributing to progressive myofiber atrophy and sarcopenia [17,18,19]. Unlike pediatric muscle vulnerability, which often reflects transient suppression of an otherwise competent anabolic environment, aging muscle decline arises from intrinsic deterioration of signaling efficiency, mitochondrial quality control, and neuromuscular integrity. Consequently, nutritional strategies targeting sarcopenia must address impaired anabolic sensitivity while also supporting mitochondrial metabolism, inflammatory regulation, and cellular proteostasis [11,20]. In Figure 1 the age-dependent regulation of skeletal muscle vulnerability is reported.

## 4. Amino Acids and mTORC1-Dependent Anabolic Signaling

**Figure 2:**
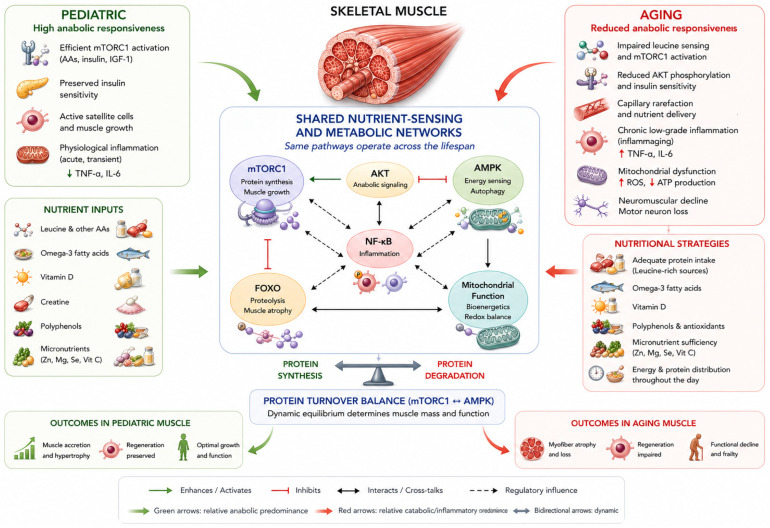
Context-dependent modulation of nutrient-sensing pathways in skeletal muscle. Skeletal muscle protein turnover is regulated by conserved nutrient-sensing and metabolic pathways integrating anabolic signaling, energy status, inflammation, and mitochondrial function. Central regulatory nodes—including mTORC1, AKT, AMPK, NF-κB, FOXO, and mitochondrial bioenergetics—form a shared network that determines the balance between protein synthesis and degradation. Dietary components such as amino acids, omega-3 fatty acids, vitamin D, creatine, polyphenols, and micronutrients converge on these pathways, modulating anabolic efficiency, inflammatory tone, and metabolic flexibility. However, the biological impact of these nutrients is not intrinsic but depends on the physiological context in which these pathways operate. In pediatric muscle, high anabolic responsiveness allows efficient activation of mTORC1-dependent protein synthesis, supported by preserved insulin sensitivity, active satellite cell function, and a controlled inflammatory environment, promoting muscle accretion and growth. In aging muscle, reduced anabolic responsiveness, characterized by impaired leucine sensing, diminished AKT–mTORC1 signaling, chronic low-grade inflammation, mitochondrial dysfunction, and neuromuscular decline, limits the effectiveness of nutrient-derived anabolic stimuli and favors protein degradation and functional decline. Thus, muscle phenotype emerges from context-dependent tuning of shared molecular pathways rather than from condition-specific mechanisms, highlighting the need for integrated nutritional strategies targeting both metabolic and functional outcomes. Arrow direction and color indicate relative pathway activity and do not imply mutually exclusive processes: green arrows denote anabolic predominance, red arrows indicate catabolic/inflammatory predominance, and bidirectional arrows indicate dynamic balance between protein synthesis and degradation. Abbreviations: AKT, protein kinase B; AMPK, AMP-activated protein kinase; GH, growth hormone; IGF-1, insulin-like growth factor-1; mTORC1, mechanistic target of rapamycin complex 1; PI3K, phosphoinositide 3-kinase.

Essential amino acids are key nutritional regulators of skeletal muscle protein synthesis. Among them, leucine plays a particularly prominent role as a signaling molecule capable of activating nutrient-sensing pathways that control translational initiation. Central to this process is mTORC1, a kinase complex that integrates signals derived from amino acid availability, insulin and IGF-1 signaling, cellular energy status, and mechanical stimuli. Activation of mTORC1 promotes phosphorylation of downstream translational regulators, including p70S6 kinase and 4E-binding protein 1, thereby stimulating ribosomal activity and increasing muscle protein synthesis [21]. Amino acid-dependent activation of mTORC1 is spatially coordinated at the lysosomal surface through the Rag-GTPase regulator complex, which recruits mTORC1 to membranes where Ras homologue enriched in brain (Rheb) enables kinase activation. Cytosolic nutrient sensors such as Sestrin2 for leucine and Cellular Arginine Sensor for mTORC1 (CASTOR1) for arginine contribute to hierarchical detection of amino acid availability, enabling skeletal muscle cells to integrate nutrient availability with anabolic signaling [22]. This system ensures that translation is activated only when adequate metabolic substrates are present to support tissue remodeling. Although the core architecture of amino acid sensing is conserved across the lifespan, its functional responsiveness differs between pediatric and aging muscle. These differences strongly influence the effectiveness of dietary protein interventions in fragile populations [23]. In Figure 2, we report the bioactive nutrients and molecular pathways involved in muscle protein turnover across fragile ages.

### 4.1. Pediatric Context: Preserved Anabolic Sensitivity

In pediatric physiology, skeletal muscle exhibits high sensitivity to anabolic stimuli. Elevated activity of the GH-IGF-1 axis, preserved insulin signaling, and high baseline protein turnover create an environment in which nutrient availability is efficiently translated into structural muscle accretion. Under adequate energy conditions, amino acid ingestion readily activates mTORC1 signaling and stimulates muscle protein synthesis [4]. However, this responsiveness can be transiently suppressed in pediatric disease states characterized by inflammation, endocrine disruption, or insufficient nutrient intake [24]. Pro-inflammatory cytokines may interfere with insulin receptor signaling and promote activation of transcription factors such as NF-κB and FOXO that favor proteolytic pathways. Likewise, sustained energy deficit activates AMPK-dependent energy sensing, which inhibits mTORC1 activity and prioritizes cellular survival processes over anabolic growth [25].

Importantly, these inhibitory influences typically act on an otherwise intact anabolic signaling framework. Consequently, restoration of energy balance, reduction of inflammatory burden, and adequate amino acid provision can rapidly re-establish physiological protein synthesis rates and support recovery of normal muscle accretion during growth [26]. However, although the anabolic responsiveness of pediatric muscle is generally preserved, clinical evidence remains limited regarding the isolated impact of amino acid supplementation on long-term functional outcomes. In most cases, improvements in protein synthesis are achieved through restoration of overall nutritional adequacy and disease control rather than through targeted amino acid interventions alone. This suggests that, in pediatric settings, amino acids act primarily within a broader context of nutritional and clinical recovery rather than as independent anabolic drivers.

Importantly, pediatric muscle vulnerability does not represent a uniform biological or clinical condition, but rather encompasses heterogeneous contexts with distinct underlying mechanisms. Undernutrition is primarily characterized by insufficient energy and protein availability, leading to impaired anabolic signaling and reduced substrate availability for growth. In contrast, chronic inflammatory diseases are associated with cytokine-mediated suppression of insulin–IGF-1 signaling and activation of proteolytic pathways [11]. In specific clinical settings, such as therapeutic ketogenic interventions, alterations in substrate utilization and hormonal regulation may further influence muscle metabolism in a context-dependent manner. These conditions differ substantially from physiological developmental states, in which transient modulation of anabolic signaling reflects normal growth dynamics rather than pathological impairment. Recognizing these distinctions is essential to avoid oversimplification and to guide more precise, context-specific nutritional strategies in pediatric populations.

### 4.2. Aging Context: Elevated Anabolic Threshold

In contrast to pediatric muscle, aging skeletal muscle demonstrates reduced responsiveness to amino acid-derived anabolic signals. This phenomenon, commonly described as anabolic resistance, reflects diminished efficiency of nutrient sensing and impaired downstream signal transduction [14].

Several mechanisms contribute to this altered responsiveness. Aging muscle exhibits reduced activation of AKT signaling, impaired mTORC1 recruitment to lysosomal membranes, and diminished ribosomal biogenesis, all of which limit translational capacity. Reduced microvascular perfusion may also impair amino acid delivery to myofibers following nutrient ingestion. In parallel, chronic low-grade inflammation and oxidative stress further interfere with insulin receptor signaling, reinforcing anabolic desensitization [27].

As a consequence, higher quantities of dietary protein or leucine may be required to achieve activation of muscle protein synthesis comparable to that observed in younger individuals. Nutritional strategies aimed at mitigating anabolic resistance therefore often emphasize increased protein density, optimized amino acid composition, and strategic distribution of protein intake across meals. Importantly, resistance exercise acts synergistically with amino acid provision by enhancing muscle perfusion and sensitizing mTORC1 signaling pathways [24]. Despite the well-established mechanistic role of amino acids, particularly leucine, in activating mTORC1 signaling, clinical evidence indicates that stimulation of muscle protein synthesis does not consistently translate into improvements in muscle strength, physical performance, or functional outcomes in older adults. Interventional studies suggest that protein supplementation alone often yields modest or variable effects, whereas more consistent benefits are observed when nutritional strategies are combined with resistance exercise. This highlights a critical dissociation between acute anabolic signaling responses and long-term functional adaptation in aging muscle. These findings underscore that overcoming anabolic resistance requires not only sufficient amino acid availability but also an integrated approach addressing physical activity, metabolic health, and underlying clinical conditions. Importantly, the clinical expression of anabolic resistance in older adults is highly heterogeneous and influenced by multiple patient-specific factors. Conditions such as frailty, multimorbidity, reduced mobility, and chronic inflammation can substantially modify the response to nutritional interventions. In addition, polypharmacy and impaired renal function may affect protein metabolism, nutrient utilization, and the safety of high-protein dietary strategies. Furthermore, preservation of muscle mass does not necessarily correspond to maintenance of muscle strength or functional capacity. Clinical outcomes such as physical performance, mobility, and independence are influenced by neuromuscular integrity and overall health status, beyond changes in lean mass alone. These considerations highlight the importance of interpreting nutritional strategies within a clinically grounded framework, in which individual variability and real-world patient complexity are taken into account.

### 4.3. Integration of Nutrient and Mechanical Signals

In vivo, activation of muscle protein synthesis depends on the coordinated interaction between nutrient-derived signals and mechanical loading. Contractile activity stimulates mechano-transduction pathways that converge on mTORC1, enhancing translational responsiveness to amino acid. This interaction explains why nutritional interventions targeting muscle health are most effective when combined with structured physical activity [28]. In pediatric populations, habitual physical activity and neuromuscular development naturally provide mechanical stimuli that complement nutrient-driven anabolic signaling [29]. In contrast, aging individuals often exhibit reduced physical activity, neuromuscular remodeling, and impaired motor unit recruitment, which further limit the anabolic response to amino acid intake [30]. Importantly, the interaction between nutrient availability and mechanical stimulation helps explain why nutritional interventions in isolation often fail to produce clinically meaningful improvements. While amino acid intake can activate intracellular signaling pathways, the absence of adequate mechanical loading may limit the translation of these signals into sustained functional adaptations. Therefore, nutritional strategies addressing muscle vulnerability should be considered within a multidimensional framework in which dietary amino acids, physical activity, and individual clinical status collectively determine the effectiveness of anabolic responses.

## 5. Lipid-Derived Bioactives: Omega-3 Fatty Acids and Ketone Metabolism

Lipids contribute to skeletal muscle regulation not only as energy substrates but also as signaling molecules capable of influencing inflammatory pathways, mitochondrial metabolism, and nutrient-sensing networks [31]. Among lipid-derived nutritional factors, long-chain omega-3 polyunsaturated fatty acids and ketone bodies have received particular attention for their ability to modulate metabolic flexibility, inflammatory tone, and mitochondrial function [32]. These lipid-derived mediators interact with transcriptional regulators and intracellular signaling pathways that influence the efficiency of anabolic responses to nutrient intake. However, their biological effects are highly dependent on the underlying metabolic context and may not uniformly translate into clinically meaningful improvements in muscle function [33].

### 5.1. Omega-3 Fatty Acids and Inflammatory-Anabolic Coupling

Long-chain omega-3 fatty acids, particularly eicosapentaenoic acid (EPA) and docosahexaenoic acid (DHA), influence skeletal muscle physiology through multiple complementary mechanisms. These fatty acids modulate inflammatory signaling, alter membrane phospholipid composition, and activate transcription factors belonging to the peroxisome proliferator–activated receptor (PPAR) family, thereby influencing lipid oxidation and mitochondrial metabolism [34]. One of the most relevant effects of omega-3 fatty acids in muscle biology is their capacity to attenuate inflammatory signaling pathways. By reducing activation of NF-κB and decreasing production of pro-inflammatory cytokines such as TNF-α and IL-6, omega-3 fatty acids can mitigate inflammatory interference with anabolic signaling pathways. Reduced inflammatory signaling may indirectly enhance the responsiveness of the insulin AKT-mTORC1 axis, supporting more efficient anabolic signaling in the presence of adequate amino acid availability [35].

In pediatric contexts characterized by chronic inflammatory disease, this anti-inflammatory action may help restore anabolic permissiveness once energy balance and protein intake are corrected. Because pediatric skeletal muscle retains a structurally intact anabolic signaling framework, reduction of inflammatory inhibition may be sufficient to allow normal growth-related muscle accretion to resume [36].

In aging muscle, the biological context differs. Omega-3 fatty acids may contribute to improved metabolic flexibility through activation of PPARα and PPARδ signaling pathways, promoting fatty acid oxidation and supporting mitochondrial biogenesis through peroxisome proliferator–activated receptor gamma coactivator 1-alpha (PGC-1α). These effects may reduce intramyocellular lipid accumulation and mitigate lipotoxic interference with insulin signaling. However, despite strong mechanistic and physiological rationale, clinical evidence regarding the impact of omega-3 supplementation on muscle mass, strength, and functional outcomes remains heterogeneous. While some studies report modest improvements in muscle protein synthesis or physical performance, others show limited or no clinically significant effects. This variability suggests that the benefits of omega-3 fatty acids are context-dependent and may require combination with other interventions, particularly resistance exercise, to achieve meaningful functional outcomes [37].

### 5.2. Ketone Bodies and Metabolic Reprogramming

Ketone bodies represent a class of lipid-derived metabolites that influence skeletal muscle metabolism during carbohydrate restriction or ketogenic interventions. During these conditions, hepatic ketogenesis increases circulating concentrations of β-hydroxybutyrate (BHB), which acts both as an oxidative substrate and as a signaling molecule [38].

Beyond its energetic role, BHB can modulate gene expression and metabolic regulation through inhibition of class I histone deacetylases, thereby promoting transcription of genes involved in oxidative stress resistance and mitochondrial maintenance. This epigenetic modulation interacts with signaling pathways involving sirtuin 1 (SIRT1) and PGC-1α, supporting mitochondrial biogenesis and improving oxidative metabolism [39].

Ketone metabolism also influences inflammatory signaling and redox balance. Experimental models suggest that BHB may attenuate NF-κB activation and reduce production of inflammatory mediators, while alterations in cellular nicotinamide adenine dinucleotide/reduced nicotinamide adenine dinucleotide (NAD^+^/NADH) ratios may enhance antioxidant defenses and mitochondrial efficiency. These mechanisms collectively position ketone bodies at the intersection of metabolic flexibility, mitochondrial maintenance, and inflammatory regulation [40].

In aging contexts characterized by mitochondrial dysfunction and metabolic inflexibility, these adaptations may partially improve cellular bioenergetics [41]. However, clinical evidence regarding the impact of ketogenic interventions on skeletal muscle mass and function remains inconsistent and highly context-dependent. Reported effects vary widely depending on protein intake, energy balance, duration of intervention, and participant characteristics.

### 5.3. Anabolic Constraints and Protein Adequacy

Despite potential metabolic benefits, ketogenic dietary interventions may introduce anabolic constraints under certain conditions. Severe carbohydrate restriction reduces circulating insulin concentrations, which normally act as a permissive signal for activation of the AKT–mTORC1 pathway [42]. If protein intake is insufficient, chronic suppression of insulin signaling may reduce translational efficiency and compromise maintenance of lean body mass [3].

Experimental findings regarding ketogenic diets and muscle mass remain heterogeneous [43]. Some studies suggest preservation of muscle function when dietary protein intake is adequate, whereas others report reductions in lean mass under conditions of insufficient protein availability [44,45].

In pediatric populations, ketogenic diets are primarily used for therapeutic purposes, particularly in refractory epilepsy and certain metabolic disorders [46]. While these interventions can provide substantial neurological benefits, prolonged ketogenic therapy in growing individuals has been associated in some cases with reduced linear growth velocity and potential alterations in lean mass development [47,48].

**Figure 3:**
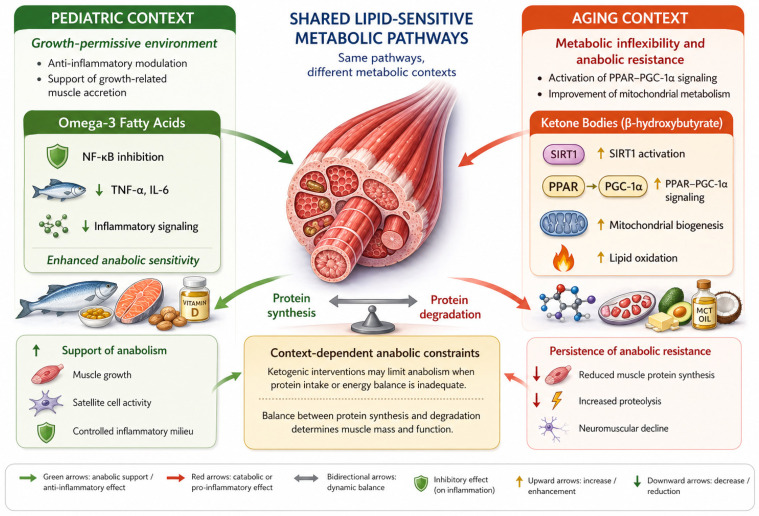
In pediatric muscle, omega-3 fatty acids contribute to a growth-permissive environment by inhibiting NF-κB signaling and reducing pro-inflammatory cytokines such as TNF-α and IL-6. This anti-inflammatory modulation supports anabolic sensitivity, facilitating efficient activation of protein synthesis and promoting muscle accretion within a physiologically intact anabolic framework. In aging muscle, ketone bodies, particularly β-hydroxybutyrate, influence metabolic pathways associated with mitochondrial function and energy utilization through activation of SIRT1 and PPAR–PGC-1α signaling. These adaptations promote lipid oxidation and mitochondrial biogenesis, partially counteracting metabolic inflexibility but not fully restoring anabolic responsiveness. Importantly, ketogenic interventions may impose context-dependent anabolic constraints, particularly when protein intake or overall energy availability is insufficient, thereby shifting the balance between protein synthesis and degradation. Overall, lipid-derived nutritional signals act on shared pathways that are differentially tuned by metabolic and physiological context, leading to distinct effects on skeletal muscle across the lifespan. Abbreviations: IL-6 interleukin-6; mTORC1 mechanistic target of rapamycin complex 1; NF-κB nuclear factor kappa B; PGC-1α peroxisome proliferator-activated receptor gamma coactivator-1α; PPAR peroxisome proliferator-activated receptor; SIRT1 sirtuin; TNF-α tumor necrosis factor-α.

Taken together (Figure 3), lipid-derived nutritional strategies illustrate how metabolic substrates can influence skeletal muscle biology through regulatory networks that integrate inflammation, mitochondrial metabolism, and nutrient sensing. Nevertheless, their clinical impact is highly context-dependent and cannot be assumed to directly translate into improvements in muscle function without consideration of overall nutritional status, protein adequacy, and physical activity.

## 6. Vitamin D and VDR-Mediated Signaling

Vitamin D exerts multiple biological effects on skeletal muscle through genomic and non-genomic mechanisms mediated by the vitamin D receptor (VDR), a nuclear transcription factor expressed in muscle fibers and satellite cells [49]. Upon binding of the active metabolite 1,25-dihydroxyvitamin D (calcitriol), VDR forms a heterodimer with the retinoid X receptor (RXR) and translocates to the nucleus, where it regulates transcription of genes involved in calcium handling, mitochondrial function, and cellular differentiation [50]. Through these transcriptional effects, vitamin D contributes to regulation of muscle contractility, metabolic homeostasis, and neuromuscular function [51]. In addition to its genomic actions, vitamin D can also exert rapid non-genomic effects through activation of intracellular signaling cascades, including mitogen-activated protein kinase pathways and modulation of intracellular calcium flux. These mechanisms may influence muscle contraction efficiency and interact with intracellular pathways that regulate protein turnover and cellular metabolism [52].

Increasing evidence indicates that vitamin D signaling interacts with metabolic and inflammatory pathways that influence muscle resilience [53]. Adequate vitamin D status has been associated with improved insulin sensitivity and more efficient activation of insulin–AKT signaling, which indirectly supports anabolic processes. Conversely, vitamin D deficiency has been linked to impaired glucose uptake, increased intramuscular lipid accumulation, and dysregulation of inflammatory signaling pathways.

### 6.1. Integration with Anabolic and Redox Signaling

Vitamin D signaling intersects with molecular networks that regulate muscle protein turnover and cellular stress responses. Activation of the VDR can modulate transcription of genes involved in oxidative stress defense, mitochondrial metabolism, and inflammatory regulation. Through these mechanisms, vitamin D contributes to stabilization of the redox environment that supports efficient anabolic signaling [51].

Experimental studies suggest that vitamin D may attenuate activation of NF κB-dependent inflammatory pathways while enhancing antioxidant defenses through interactions with redox-sensitive transcription factors. This regulatory role positions vitamin D at the interface between endocrine regulation, inflammatory signaling, and mitochondrial metabolism [53].

Importantly, these effects appear primarily permissive rather than directly anabolic. Vitamin D does not typically act as a primary driver of muscle hypertrophy but instead contributes to maintaining the physiological conditions required for efficient responsiveness to other anabolic stimuli such as amino acids and mechanical loading [54].

### 6.2. Pediatric Context: Growth-Permissive Regulation

In pediatric populations, vitamin D plays a fundamental role in musculoskeletal development. Adequate vitamin D status supports calcium homeostasis, skeletal mineralization, and neuromuscular coordination, all of which indirectly influence muscle performance and physical development [55].

Vitamin D deficiency during childhood has been associated with reduced muscle strength, impaired motor performance, and delayed achievement of developmental milestones. These effects are likely mediated through alterations in calcium-dependent excitation–contraction coupling and through indirect influences on endocrine signaling pathways involved in growth [56].

**Figure 4:**
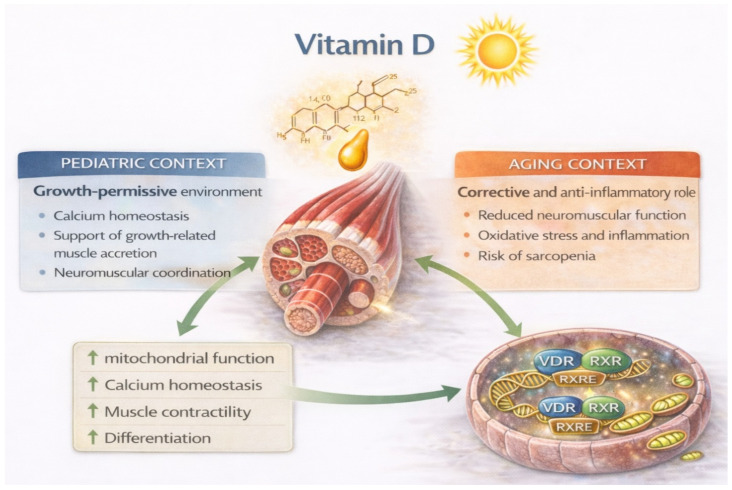
Vitamin D-VDR signaling in skeletal muscle regulation. The active vitamin D metabolite calcitriol binds the vitamin D receptor (VDR), which forms a heterodimer with the retinoid X receptor (RXR) and regulates transcription of target genes through vitamin D response elements (VDREs). VDR signaling promotes mitochondrial function, calcium homeostasis, muscle contractility, and cellular differentiation while reducing NF-κB-mediated inflammatory signaling. These mechanisms contribute to the maintenance of an anabolic-permissive environment that supports efficient responsiveness of skeletal muscle to anabolic stimuli.

However, in the context of normal pediatric physiology characterized by preserved anabolic signaling capacity, vitamin D primarily acts as a growth-permissive factor rather than as a direct stimulator of muscle protein synthesis [57]. Correction of vitamin D deficiency therefore restores physiological conditions required for optimal musculoskeletal development but does not independently drive muscle hypertrophy beyond normal growth trajectories (Figure 4) [58].

### 6.3. Aging Context: Corrective and Anti-Inflammatory Role

In older adults, hypovitaminosis D is highly prevalent due to a combination of reduced cutaneous synthesis, decreased dietary intake, and alterations in renal activation of vitamin D metabolites. Aging muscle may also exhibit reduced expression of the vitamin D receptor, potentially limiting transcriptional responsiveness to circulating vitamin D [59]. Low vitamin D status has been consistently associated with impaired muscle strength, reduced physical performance, and increased risk of falls in older populations. Mechanistically, vitamin D insufficiency may exacerbate chronic inflammatory signaling and oxidative stress, thereby contributing to impaired metabolic regulation within skeletal muscle [51]. Restoration of adequate vitamin D status may improve neuromuscular function and reduce inflammatory interference with metabolic signaling pathways. Nevertheless, clinical trials indicate that vitamin D supplementation alone has limited capacity to reverse established sarcopenia [53]. Improvements in muscle function appear most evident in individuals with baseline deficiency and when vitamin D correction is combined with adequate protein intake and resistance exercise [60]. Taken together, vitamin D functions as an endocrine regulator that supports the physiological substrate upon which anabolic signaling operates. In pediatric muscle, adequate vitamin D status contributes to growth-permissive conditions for musculoskeletal development. In aging muscle, correction of deficiency may help mitigate inflammatory and metabolic constraints that contribute to functional decline [61]. These observations further highlight the distinction between molecular effects on muscle metabolism and clinically meaningful improvements in functional outcomes, reinforcing the need for integrated intervention strategies.

## 7. Redox Modulation: Polyphenols, Nrf2, and Mitochondrial Resilience

Cellular redox balance plays a critical role in maintaining skeletal muscle integrity across the lifespan. Reactive oxygen species (ROS) are continuously generated during mitochondrial respiration and cellular metabolism, acting both as damaging agents when present in excess and as signaling intermediates that regulate adaptive responses to metabolic stress and physical activity. The ability of skeletal muscle to maintain redox homeostasis therefore represents a key determinant of metabolic efficiency, mitochondrial quality control, and protein turnover [62].

Among dietary bioactive compounds, polyphenols and carotenoids have attracted increasing attention for their capacity to modulate oxidative stress responses and mitochondrial function. Rather than acting simply as direct antioxidants, many of these compounds influence intracellular signaling pathways that regulate cellular stress adaptation, particularly through activation of nuclear factor erythroid 2–related factor 2 (Nrf2) [63].

Under basal conditions, Nrf2 is bound to the cytosolic protein Kelch-like ECH-associated protein 1 (Keap1), which targets the transcription factor for ubiquitin-mediated degradation. Oxidative or electrophilic stimuli modify cysteine residues within Keap1, allowing Nrf2 stabilization and nuclear translocation [64]. Once in the nucleus, Nrf2 binds to antioxidant response elements and stimulates transcription of genes involved in glutathione synthesis, NADPH regeneration, heme oxygenase-1 expression, and mitochondrial protection [65]. Through this transcriptional program, Nrf2 activation enhances cellular antioxidant capacity, preserves mitochondrial membrane potential, and limits ROS-mediated activation of inflammatory and proteolytic pathways [66]. These mechanisms place Nrf2 signaling at the intersection of oxidative stress regulation, mitochondrial maintenance, and metabolic resilience.

### 7.1. Hormetic Signaling and Mitochondrial Biogenesis

Beyond induction of antioxidant defenses, several dietary polyphenols including resveratrol, quercetin, and epigallocatechin gallate can influence mitochondrial metabolism through activation of signaling pathways involving SIRT1 and PGC-1α. Activation of this SIRT1-PGC-1α axis promotes mitochondrial biogenesis, enhances oxidative phosphorylation capacity, and improves metabolic flexibility [67].

These effects illustrate the concept of nutritional hormesis, whereby mild electrophilic or oxidative signals induced by dietary phytochemicals stimulate adaptive cellular responses that strengthen stress resistance mechanisms. In this context, polyphenols act less as direct radical scavengers and more as modulators of cellular signaling networks that regulate mitochondrial turnover and redox balance [68]

Reactive oxygen species themselves also exhibit dual roles in skeletal muscle physiology. While excessive reactive oxygen species (ROS) can impair insulin signaling and promote proteolysis, moderate ROS production functions as an important signaling mechanism during exercise, contributing to mitochondrial biogenesis and metabolic adaptation [69]. Consequently, excessive supplementation with high-dose antioxidant compounds may blunt these adaptive responses, reinforcing the importance of dietary rather than pharmacological redox modulation [70].

### 7.2. Pediatric Context: Oxidative Stress and Recovery of Anabolic Signaling

In pediatric fragile populations, oxidative stress is often secondary to disease-associated inflammation, infection, or metabolic disturbances rather than to intrinsic cellular aging [71]. Elevated ROS production in these contexts can interfere with endocrine and nutrient-dependent signaling pathways that regulate muscle growth.

Oxidative stress may impair insulin and IGF-1 signaling and promote activation of transcription factors that favor proteolytic pathways. By enhancing antioxidant defenses and improving mitochondrial function, dietary polyphenols may help reduce redox-mediated interference with anabolic signaling pathways [72]. When combined with adequate nutritional intake and control of inflammatory disease activity, this redox stabilization may contribute to restoration of physiological growth-related muscle accretion [73].

### 7.3. Aging Context: Redox Decline and Mitochondrial Dysfunction

In aging skeletal muscle, redox imbalance emerges as a central component of the biological processes contributing to sarcopenia. Progressive mitochondrial dysfunction leads to increased production of ROS, accumulation of oxidatively damaged proteins, and impairment of cellular quality control systems [74].

This chronic oxidative stress environment contributes to activation of inflammatory pathways and may further interfere with insulin and IGF-1 signaling, thereby reinforcing anabolic resistance. Reduced mitochondrial turnover and impaired mitophagy exacerbate these processes, promoting accumulation of dysfunctional organelles and further compromising metabolic efficiency [75].

Clinical evidence further supports the relevance of oxidative stress in older populations. In hospitalized older adults, alterations in oxidative stress markers and adipokine profiles have been shown to vary according to nutritional status, suggesting a close interaction between redox imbalance, metabolic regulation, and clinical vulnerability. These findings provide translational support linking mechanistic redox dysregulation to biomarker-based clinical phenotypes in aging muscle [76].

Polyphenol-rich dietary patterns may help mitigate these mechanisms by enhancing endothelial function, stimulating mitochondrial biogenesis, and attenuating inflammatory signaling pathways. Nevertheless, current evidence suggests that the beneficial effects of polyphenols are most pronounced when they are integrated within balanced dietary patterns and combined with regular physical activity, which remains a primary stimulus for mitochondrial adaptation in skeletal muscle [77].

## 8. Micronutrients and Mitochondrial Bioenergetics

Micronutrients play essential roles in skeletal muscle metabolism by supporting enzymatic reactions involved in energy production, antioxidant defense, and protein synthesis. Unlike amino acids or lipid-derived bioactives, micronutrients rarely act as direct stimulators of anabolic signaling. Instead, their primary contribution lies in maintaining the metabolic and bioenergetic conditions required for efficient muscle function and tissue remodeling [78].

Several minerals—including iron, magnesium, zinc, and selenium—are integral components of mitochondrial enzymes and redox-regulating proteins. Through these roles, micronutrient availability influences mitochondrial respiration, ATP production, and oxidative stress regulation, all of which contribute to maintenance of skeletal muscle integrity. Deficiencies in these nutrients may therefore compromise muscle metabolism indirectly by impairing cellular bioenergetics and increasing vulnerability to oxidative stress [79].

### 8.1. Pediatric Context: Growth-Dependent Bioenergetic Demand

During childhood and adolescence, rapid tissue growth and high metabolic activity increase the physiological demand for micronutrients involved in mitochondrial energy production and cellular proliferation. Adequate availability of these minerals is essential to sustain the energetic requirements of muscle protein synthesis and developmental remodeling [80].

Iron plays a central role in mitochondrial respiration through its incorporation into cytochromes and iron–sulfur clusters that support electron transport chain activity. Iron deficiency can therefore reduce oxidative phosphorylation efficiency and limit ATP production required for growth-related anabolic processes [81].

Magnesium acts as a cofactor for numerous enzymes involved in energy metabolism and stabilizes ATP molecules required for kinase activity within intracellular signaling pathways [82]. Zinc contributes to ribosomal function and protein synthesis while also participating in regulation of endocrine pathways influencing growth [83]. Selenium supports antioxidant defense through its incorporation into selenoproteins such as glutathione peroxidases, which protect mitochondrial membranes from oxidative damage [84].

In pediatric fragile populations, deficiencies in these micronutrients may arise from inadequate dietary intake, malabsorption, chronic disease, or increased metabolic demands [85]. Correction of these deficiencies can restore metabolic permissiveness and support physiological muscle development by improving mitochondrial function and cellular energy availability [86].

### 8.2. Aging Context: Mitochondrial Reserve and Hidden Deficiency

In older adults, the physiological role of micronutrients shifts from supporting growth to preserving mitochondrial reserve capacity and metabolic resilience. Aging skeletal muscle exhibits reduced mitochondrial density, impaired oxidative metabolism, and increased susceptibility to oxidative stress [87]. Subclinical deficiencies of micronutrients may therefore exacerbate these age-related alterations.

Reduced dietary intake, impaired nutrient absorption, and polypharmacy commonly contribute to micronutrient insufficiency in aging populations. Even mild deficiencies may compromise mitochondrial enzyme activity, increase production of reactive oxygen species, and promote activation of energy-sensing pathways associated with metabolic stress [88].

This bioenergetic constraint may reinforce anabolic resistance by limiting the cellular energy available for protein synthesis and by favoring catabolic signaling pathways that prioritize maintenance of cellular homeostasis. Consequently, adequate micronutrient intake contributes to preservation of metabolic flexibility and mitochondrial function in aging skeletal muscle [89].

**Figure 5:**
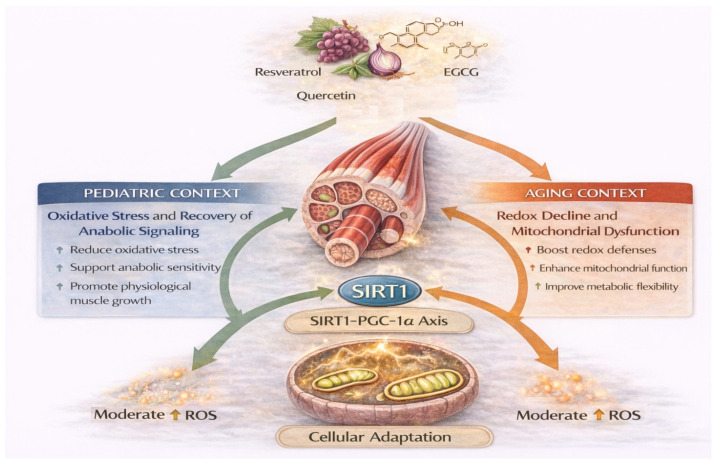
Hormetic signaling and mitochondrial adaptation induced by dietary polyphenols. Polyphenols such as resveratrol, quercetin, and epigallocatechin gallate activate adaptive stress-response pathways including the SIRT1–PGC-1α axis, promoting mitochondrial biogenesis and metabolic flexibility. Moderate reactive oxygen species (ROS) act as signaling mediators that stimulate mitochondrial adaptation, whereas excessive oxidative stress may impair anabolic signaling. The physiological impact of redox modulation differs between pediatric muscle, where oxidative stress may disrupt growth-related signaling, and aging muscle, where mitochondrial dysfunction and redox imbalance contribute to sarcopenia.

Importantly, current evidence suggests that restoring physiological micronutrient sufficiency is generally sufficient to support muscle metabolism. Supra-physiological supplementation has not consistently demonstrated additional benefits for muscle mass or strength and may in some cases disrupt normal redox balance [90]. Nutritional strategies targeting muscle health in older adults therefore emphasize balanced dietary patterns rather than isolated high-dose supplementation. In Figure 5 we reported the Hormetic signaling and mitochondrial adaptation induced by dietary polyphenols.

## 9. Conclusions and Future Directions

Muscle vulnerability at the extremes of the human lifespan reflects two biologically distinct regulatory states rather than a single pathological continuum. In pediatric populations, impaired muscle development generally arises from disruption of an otherwise competent anabolic environment that normally supports rapid tissue growth [13]. In contrast, muscle decline in older adults emerges from progressive deterioration of signaling efficiency, mitochondrial function, and neuromuscular integrity, leading to reduced responsiveness to anabolic stimuli and increased susceptibility to catabolic remodeling [91].

Across both contexts, nutritional factors influence skeletal muscle biology through a conserved regulatory architecture centered on nutrient sensing, inflammatory signaling, and mitochondrial metabolism [78]. Amino acids regulate translational pathways governing protein synthesis, lipid-derived mediators modulate inflammatory tone and metabolic flexibility, vitamin D contributes to endocrine and redox regulation, polyphenols influence oxidative stress responses and mitochondrial resilience [92].

However, the biological impact of these nutritional signals is strongly conditioned by developmental stage and by the integrity of underlying anabolic signaling networks. Importantly, improvements in molecular or biochemical markers of muscle metabolism do not necessarily correspond to clinically meaningful benefits. Outcomes such as muscle strength, physical performance, mobility, and independence represent critical endpoints that are not consistently predicted by changes in signaling pathways or protein synthesis rates alone.

A critical challenge emerging from current evidence is the dissociation between mechanistic plausibility and clinical effectiveness. While numerous nutritional interventions modulate molecular pathways involved in muscle protein turnover and metabolic regulation, their effects on clinically meaningful outcomes—such as muscle strength, physical performance, mobility, and functional independence—remain variable and often inconsistent. This gap underscores the limitations of extrapolating mechanistic findings into clinical recommendations without robust outcome-based evidence. In pediatric fragile populations, nutritional interventions primarily aim to restore physiological conditions required for growth by correcting energy imbalance, balanced protein provision, and correction of micronutrient deficiencies within the context of disease management [93].

In aging individuals, effective interventions must address anabolic resistance through integrated approaches combining optimized protein intake, resistance exercise, and modulation of inflammatory and metabolic constraints [94].

From a translational perspective, current evidence supports a stratified interpretation of nutritional interventions. Strategies with consistent clinical support include adequate protein intake combined with resistance exercise and correction of overt micronutrient deficiencies.

**Figure 6:**
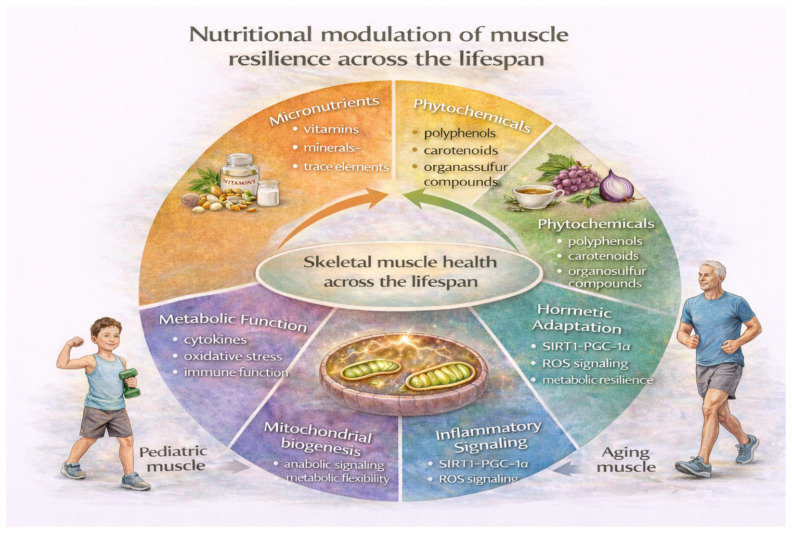
Nutritional modulation of skeletal muscle resilience across the lifespan. Schematic overview of the principal nutritional and metabolic factors influencing skeletal muscle physiology from childhood to aging. Micronutrients, phytochemicals, and hormetic signaling pathways contribute to maintenance of mitochondrial function, redox balance, inflammatory regulation, and metabolic flexibility. These mechanisms support muscle development in pediatric physiology and help preserve mitochondrial resilience and functional capacity during aging. Abbreviations: ROS, reactive oxygen species; SIRT1 sirtuin 1; PGC-1α, peroxisome proliferator-activated receptor gamma coactivator-1 alpha.

In contrast, several bioactive compounds—including omega-3 fatty acids, vitamin D beyond deficiency states, and polyphenols—show promising mechanistic and physiological effects but yield heterogeneous or modest results in clinical settings. Similarly, emerging approaches within precision nutrition remain largely hypothesis-generating and require validation through well-designed interventional studies. Future research should move beyond isolated nutrient supplementation and prioritize well-designed interventional studies focusing on functional endpoints rather than solely molecular or biochemical markers. Greater emphasis on patient stratification, including metabolic status, inflammatory burden, and baseline functional capacity, will be essential to identify subpopulations most likely to benefit from targeted interventions. Ultimately, advancing musculoskeletal health across the lifespan will require a shift toward precision nutrition frameworks that align dietary strategies with the biological context of muscle vulnerability (Figure 6). Bridging the gap between mechanistic insight and clinical effectiveness remains the central challenge for translating nutritional science into meaningful improvements in muscle function and quality of life.

## Data Availability

Not applicable. This manuscript is a narrative review of the literature and did not involve the generation or analysis of original datasets. Consequently, no datasets are available for sharing.
